# Measuring small-scale plasma irregularities in the high-latitude E- and F-regions simultaneously

**DOI:** 10.1038/s41598-023-38777-4

**Published:** 2023-07-18

**Authors:** Magnus F. Ivarsen, Jean-Pierre St-Maurice, Glenn Hussey, Andres Spicher, Yaqi Jin, Adam Lozinsky, Lindsay V. Goodwin, Draven Galeschuk, Jaeheung Park, Lasse B. N. Clausen

**Affiliations:** 1 Department of Physics, University of Oslo, Oslo, Norway; 2 Department of Physics and Engineering Physics, University of Saskatchewan, Saskatoon, SK Canada; 3 Department of Physics and Astronomy, University of Western Ontario, London, ON Canada; 4 Department of Physics and Technology, UIT the Arctic University of Norway, Tromsø, Norway; 5 Center for Solar-Terrestrial Research, New Jersey Institute of Technology, Newark, NJ USA; 6 Korea Astronomy and Space Science Institute, Taejon, South Korea; 7 Department of Astronomy and Space Science, Korea University of Science and Technology, Taejon, South Korea

**Keywords:** Space physics, Aurora, Plasma physics, Space physics, Aurora

## Abstract

The ionosphere, Earth’s space environment, exhibits widespread turbulent structuring, or plasma irregularities, visualized by the auroral displays seen in Earth’s polar regions. Such plasma irregularities have been studied for decades, but plasma turbulence remains an elusive phenomenon. We combine scale-dependent measurements from a ground-based radar with satellite observations to characterize small-scale irregularities simultaneously in the bottomside and topside ionosphere and perform a statistical analysis on an aggregate from both instruments over time. We demonstrate the clear mapping of information vertically along the ionospheric altitude column, for field-perpendicular wavelengths down to 1.5 km. Our results paint a picture of the northern hemisphere high-latitude ionosphere as a *turbulent system* that is in a constant state of growth and decay; energy is being constantly injected and dissipated as the system is continuously attempting an accelerated return to equilibrium. We connect the widespread irregularity dissipation to Pedersen conductance in the E-region, and discuss the similarities between irregularities found in the polar cap and in the auroral region in that context. We find that the effects of a conducting E-region on certain turbulent properties (small-scale spectral index) is near ubiquitous in the dataset, and so we suggest that the electrodynamics of a conducting E-region must be considered when discussing plasma turbulence at high latitudes. This intimate relationship opens up the possibility that E-region conductivity is associated with the *generation* of F-region irregularities, though further studies are needed to assess that possibility.

## Introduction

The properties of plasma in the high latitude ionosphere are determined to a large degree by the interaction between Earth’s magnetosphere and the solar wind^1^. This ultimate injection of energy from the solar wind is made most manifest in the ionosphere through particle precipitation and the display of aurora that it triggers. Impacting precipitating particles produce electric fields and pass electrical currents that account for the slowing action of the ionosphere on the solar wind. Locally, strong electric fields, plasma convection, and sharp gradients in the plasma density work in tandem to create instabilities^2,3^, which may lead to turbulence and plasma irregularities.

High-latitude irregularities mostly evolve in directions perpendicular to the nearly vertical magnetic field lines, owing to a fast and efficient field-aligned (vertical) transport of plasma, from which it has been shown that an individual plasma irregularity structure should have a very long vertical wavelength^4–7^. As a result, ionospheric plasma irregularities are often described in terms of two-dimensional turbulence, with an irregularity structure’s field-perpendicular wavelength essentially denoting the size of the irregularity. At some point turbulent information is no longer mapped between the bottomside (E-region) and topside (F-region) ionosphere. It has been assumed that the perpendicular scale-size of such non-mapping irregularities is well above 1 km^8^, though a recent paper^9^ presented in an appendix back-of-the-envelope calculations which indicate that scales well *below* 1 km easily map between peak E-region and topside F-region altitudes.

Systematic studies of the ionosphere’s entire altitude column have been few, owing to the difficulty of obtaining data with good coverage in both horizontal geomagnetic coordinates and in altitude. While in situ measurements made by spacecraft such as satellites and rockets in the past have spanned virtually all altitudes, such measurements are inherently *local*, and there is no way to directly probe any direction other than ‘forward’ in the spacecraft frame of reference. Spacecraft make one-dimensional slices through ionospheric plasma, and assume that information present in the perpendicular dimensions is projected onto one dimension: a useful assumption that nevertheless can be problematic^10^. Despite these limitations, spacecraft have proved excellent tools to study a wide variety of plasma physical phenomena in the ionosphere on scales ranging from $\sim 1$ cm up to 100 km^11–15^.

Ground-based experiments using radar can probe extended altitude ranges, often spanning parts of both the E- and F-regions, and can monitor large volumes of plasma simultaneously. The large Super Dual Auroral Radar Network (Superdarn) can effectively observe the large-scale convection of plasma in the ionosphere, by analyzing a multitude of individual irregularities^16^, and incoherent scatter radars (ISRs), such as the European Incoherent Scatter Scientific Association (eiscat)^17^ observe the motions of a myriad individual electrons simultaneously, yielding scale-dependent information about the growth of irregularities^18^. Recently, ISR technology was used to reconstruct the ionospheric irregularity field, aggregating altitude-dependent multi-point plasma measurements^19^.

A recent study based on the coherent scatter radar icebear 3D developed a method to simultaneously measure plasma at a large continuous scale interval covering scales between 1.5 and 25 km^20^. The method correlates the positions of individual 3-m Farley–uneman (FB) waves in the E-region, and produces a spatial power spectrum of their tendency to cluster in space, by application of Monte-Carlo based tools adapted from cosmological galaxy surveys. The resulting clustering spectrum was demonstrated to match the structuring, or *filamentation* of the aurora above, as measured by satellites orbiting in the topside F-region^20^.

### Plasma turbulence and dissipation

As both a source of free energy, and a strong modulator of electric fields, the high-energy particle precipitation of the aurora is a crucial ingredient in the production of high-latitude plasma irregularities^2^. In the E-region, the nightside aurora is directly associated with great enhancements in ionospheric conductivity^21–23^, and generally consists of electrons with high kinetic energy^24,25^. The free energy provided, or *injected*, by collisions between precipitating particles and the neutral atmosphere in the aurora rightly means that the whole system at times is extremely structured, and turbulence is widespread, extending down to cm-scale^26,27^. Then, usual avenues of dissipation such as heating by particle impact, chemical effects and Joule heating are not the only processes that need to be taken into account for the dissipation of energy in the ionosphere-atmosphere system. While turbulence is triggered in order to smooth out gradients in velocity, density, and temperature, it also becomes a possibly significant channel through which energy is being *dissipated*.

**Figure 1:**
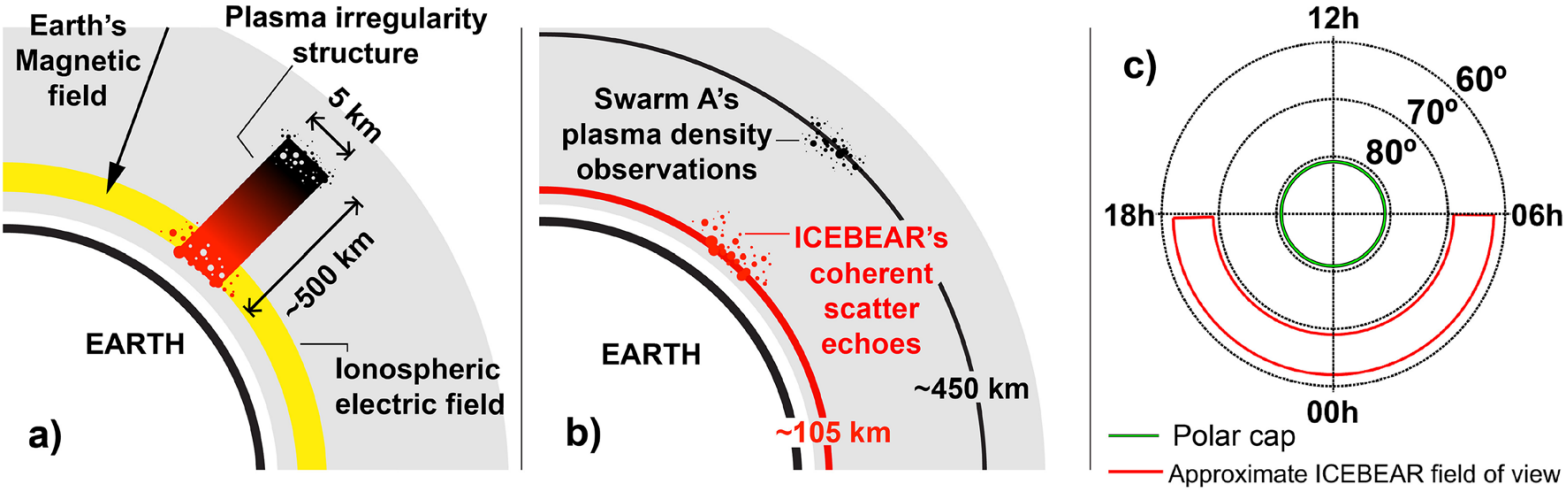
Schematic presentations of the ionosphere. Panel (**a**) shows a schematic slice through the ionosphere, displaying a plasma irregularity structure observed somewhere in the F-region, with a field-perpendicular (horizontal) wavelength of 5 km, and with a field-aligned (vertical) wavelength on the order of 500 km. Also indicated is the direction of Earth’s magnetic field in the northern hemisphere and the ionospheric electric field at the E-region peak. (We take the opportunity here to point out that Earth’s northern geomagnetic pole is in fact a magnetic south pole, hence the direction of the black vector in Panel (**a**). Panel (**b**) shows a schematic slice through Earth’s ionosphere, with Swarm A’s orbit displayed as a solid black line, and the central altitude at which icebear 3D detects coherent scatter echoes displayed as a solid red line. The figure shows that Swarm A samples the topside F-region while icebear samples the ionosphere near the E-region peak. Panel (**c**) shows a schematic of the high-latitude ionosphere, from a vantage point directly above Earth’s magnetic north pole. In this representation, the Sun is located towards 12 h (noon) and dawn (06 h) is to the right, while circles of constant magnetic latitude are indicated. A working definition of the polar cap (poleward of 82$^\circ$ mlat) and icebear’s approximate field of view are shown in green and red line respectively, while Swarm A’s orbit yields a global coverage.

The energy dissipation from density irregularities happens through two main channels^28^. One, typically studied in Fourier space, is the transmission of energy from one scale to another^29,30^. Here, one talks about mode-coupling as a mechanism that leads to a ‘cascade’ from larger to smaller scales. At some point the cascade would reach scales for which molecular processes will take care of the dissipation. Such molecular processes are described by plasma diffusion. One intriguing point originally raised by^8^ and brought back by^31^ is that when unstable structures are introduced in the F-region, the presence of diffusion is greatly accelerated if the plasma can connect to a conducting E region. This requires two conditions, the first being related to scales involved. The scales must be small enough for diffusion to affect them and also be large enough to be able to map down to the E-region. The other condition is simply that there be a conducting E-region to begin with. Panel a) of Fig. 1 illustrates these criteria schematically; when an individual electrostatic irregularity structure in the F-region (black and red rectangle) is able to connect to a conducting E-region (yellow band), fast ambipolar diffusion causes the irregularity structure to dissipate. If the *field-perpendicular* wavelength of this irregularity structure (5 km in Fig. 1a) is too short, the *field-aligned* wavelength will subsequently be too short for the structure to map down to the E-region. Whether any given irregularity structure is able to map down to the E-region is dependent on ionospheric conductivities, but a 5 km structure observed at around 450 km altitude should always map down to the E-region^9^. In Fourier space, the turbulent cascade associated with a dissipating electrostatic irregularity structure accelerates at some intermediate scale, and the irregularity spectrum steepens as a result of increased dissipation at smaller scales^9,12,13^. As mentioned above, whether the irregularity in question is able to connect to the E-region is dependent on its field-perpendicular wavelength. The question of energy injection and subsequent dissipation has been addressed by various authors in the recent past^3,9,32,33^, and remains an elusive topic.

In this study, we present new results from the active high-latitude region of the ionosphere, where we demonstrate that turbulent dissipation of plasma irregularities is ubiquitous to the extent that one cannot talk about energy injection (leading to growth) and dissipation (leading to decay) as being distinct phenomena in ionospheric plasma. While energy is being pumped into the ionosphere-atmosphere system at irregular intervals, it is also effectively being dissipated away. We demonstrate that small-scale information, in this case plasma turbulence, map along Earth’s field lines between the E- and F-regions for scales down to 1.5 km. We show this by three direct conjunctions between the icebear 3D radar and ESA’s Swarm satellites, yielding simultaneous measurements of turbulent structuring in the E- and F-regions. We go on to perform a statistical analysis to extensive datasets from both instruments to reinforce the results derived from conjunctions. We discuss the similarities between irregularities in the polar cap and those in the auroral region, where E-region conductivity dynamics is the main feature shared by both regions, indicating that the role played by the electrical conductivity of the ionosphere is greater than has hitherto been thought: we suggest that there is an intrinsic connection between turbulence in the F-region on the one hand and E-region conductance on the other.

## Methods

The two main datasets used in the present study are illustrated schematically in Panel b of Fig. 1. The Swarm A satellite from the European Space Agency’s Swarm mission^34^ orbits through the ionosphere’s F-region (solid black line), while the Canadian icebear 3D coherent scatter radar^35^ surveys the ionosphere’s E-region (solid red line).

In the F-region, we rely in the present study on the high-resolution plasma density observations from the faceplate of the Electric Field Instrument (EFI)^36^ on board Swarm. The Swarm satellites’ orbits cover all magnetic local times in a 131-day cycle, at an altitude of around 500 km. The 16 Hz Advanced Plasma Density calculations are produced by the current through the satellite’s faceplate in conjunction with the on-board Langmuir probe. We use these observations to calculate Power Spectral Density (psd), yielding density spectra. We rely on detection and characterization of *steepening spectra*, spectra that exhibit a break-point and a subsequent drop in power, using a method that is documented extensively in^9,31^. In short, the analysis entails automatic calculation and detection of steepening plasma density spectra. For the density spectra themselves, we use a method based on Welch’s psd^37,38^. To identify the presence of spectral breakpoints, we first consider the psd to conform to a dual-slope power law, where slope, also called spectral index, refers to the slope of a log-log linear fit of power versus frequency. To compute spectral indices, we fit a piece-wise linear Hermite function to the logarithm of the spectrum^39^. If a spectral fit to the log of a spectrum shows a numerical difference between the first and second spectral slopes greater than 0.8, with the second slope being steepest, we infer a spectral break associated with steepening. As discussed in^9^, the spectral break needs to be found between the frequencies of 0.19 Hz and 6.5 Hz, corresponding to along-track spatial scales between 39.9 and 1.2 km, respectively. We perform the preceding analysis on 60-s segments of plasma density sampled at high latitudes, with a cadence of 5 s, meaning the spectra overlap extensively. The 16 Hz original sampling frequency is then reduced to a timeseries of density spectra with a 5-s resolution. From this analysis, we extract and store the second spectral index (small-scale spectral index) for the steepening spectra.

**Figure 2:**
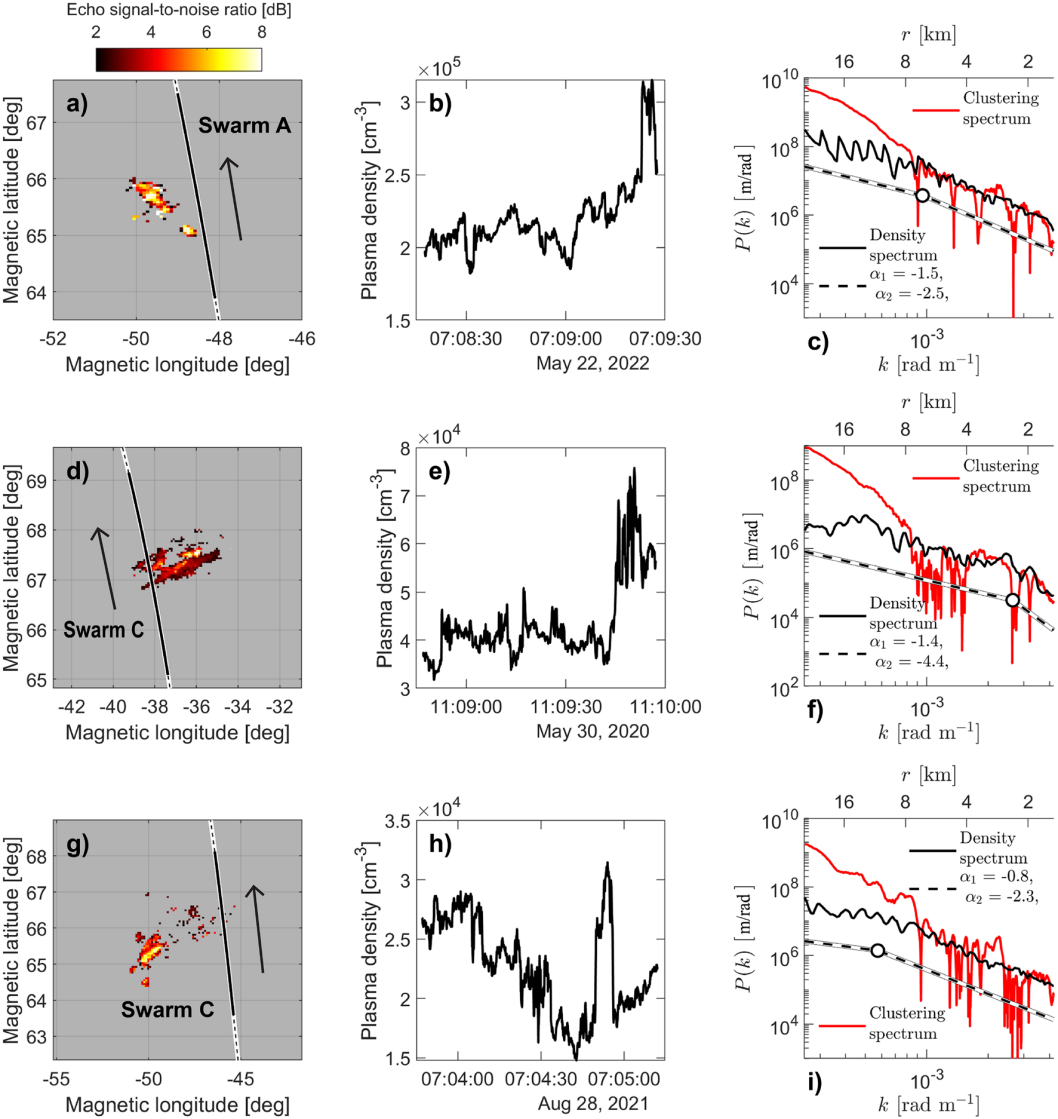
Three conjunctions between Swarm satellites and icebear 3D. Panels (**a**,**d**,**g**) show icebear 3D echo signal-to-noise ratio (SNR) in a colorscale and Swarm orbit in black line. The solid black line corresponds to the stretch of orbit used for the density measurements in panels (**b**,**e**,**h**). Panels (**c**,**f**,**i**) compare the E-region clustering spectra (solid red line) with the F-region density spectra (solid black line), normalized to the same rms. Black dashed lines show a double-slope fit of the F-region density spectra, with a white circle denoting the location of the break-point. In all nine panels, black-colored data stem from the Swarm satellites (the 16 Hz density dataset), and red-colored data stem from icebear. Note that panels (**a**,**d**,**g**) are all wholly within icebear’s field-of-view — echo detection is generally sparse at any given time.

The other dataset consists of radio echoes from 3-m density gradients produced by the Farley–Buneman instability^40,41^. icebear, or The Ionospheric Continuous-wave E-region Bistatic Experimental Auroral Radar, located in Saskatchewan, Canada, operates at a frequency of 49.5 MHz^35^. It was recently reconfigured to detect echoes with an unprecedented spatial resolution in both altitude and azimuth, along with a wide field of view, resulting in the icebear 3D dataset^42,43^. The results shown in the present study are based on 114 million individual E-region echoes, that are projected on a spherical shell 105 km above Earth’s surface. Motivated by how these echoes appear to cluster on that shell^44^, a new method of analysis applies two-point correlation statistics to echo populations, which allows the estimate of an auto-correlation function for the two-dimensional *echo density distribution*^20^. The two dimensions in question are those forming a plane perpendicular to Earth’s magnetic field lines. The spatial correlations in turn yield a novel method to estimate the power spectral density of the apparent *clustering* of FB waves in the E-region ionosphere. It is important here to note that while icebear’s detected irregularities have spatial scales of 3 m, we are relying on the fact that individual coherent scatter echoes trace the greater field of plasma irregularities in the E-region. This field was shown to correspond remarkably well to the small-scale structuring in the currents produced by the aurora, on all scales between 1.5 and 25 km^20^.

Except for three auspicious conjunctions that we shall soon describe, the time periods for the two datasets used in the present study do not overlap. The 16 Hz Advanced plasma density dataset contains only sporadic high-latitude observations after 2020, while the icebear 3D datasets have no observations prior to 2020. Swarm 16 Hz density measurements increased in 2022, but nominal auroral electrojet indices are not yet available for that year. The non-overlapping aggregated results are nevertheless consistent with three direct conjunctions between the instruments, and we show that both datasets roughly conform to predictable long term (solar cycle) variations. The Swarm (F-region) dataset stretches from late 2014 through 2019, from which we have extracted 510,000 steepening spectra. The icebear dataset stretches from 2020 through 2021, and consists of 7350 spectra, where each spectrum is based on distinct populations of echoes, with each population containing between 1000 and 500,000 echoes.

For the comparison between Swarm and icebear we compare observations in the nightside auroral region, which constitutes icebear’s operating times and approximate field of view. (icebear operation has largely been limited to the nightside due to cost efficiency—icebear’s field-of-view during daytime is usually equatorward of the dayside aurora.) We define the nightside auroral region to be confined between 60$^\circ$ and 70$^\circ$ magnetic latitude (mlat), and between 18 and 06 h magnetic local time (mlt), where we use the altitude-adjusted corrected geomagnetic coordinates system to calculate geomagnetic coordinates^45^. While we use both *in-situ* and ground-based instruments to characterize the auroral region, we also analyze Swarm-based F-region measurements from the polar cap (poleward of 82$^\circ$ magnetic latitude, solid green line in Fig. 1c). In the present study, we use only data from the northern hemisphere, where icebear is located, but the *in-situ* results reported herein are comparable in the southern hemisphere^9^, Figure 2 therein.

## Results

Figure 2 introduces the two instruments used and presents the only three conjunctions identified between icebear and a Swarm satellite. Each row corresponds to a conjunction while each column shows the different measurements made by the two instruments. The left-hand column (panels a, d, and g) show the icebear 3D echo distribution, with signal-to-noise ratio (SNR) expressed in dB with a colorscale, and the orbital trajectory of a Swarm satellite is shown with a solid black line. The middle column (panels b, e, and h) then shows plasma density time-series as observed by Swarm during the conjunction in question, a 75-s period in each conjunction.

The right-hand column (panels c, f, and i) shows a direct comparison between the F-region density spectrum (black, measured by Swarm) and the E-region clustering spectrum (red, measured by icebear). The lower *x*-axis shows wavenumber *k*, and the upper *x*-axis shows *r*, the spatial scale associated with each wavenumber: $k~=~2\pi /r$. icebear’s clustering spectrum is an inherent spatial spectrum, and is naturally expressed as a function of *k*. In-situ spectra from Swarm, on the other hand, are temporal spectra derived from a Fourier analysis of time-series. These are converted to spatial spectra by assuming the plasma to be stationary with respect to the spacecraft. As the orbital speed of a satellite in low-Earth-orbit is around 7.6 km/s, this assumption will usually hold true, but might break down for extremely fast plasma convection (on the order of a few km/s). For such temporal-to-spatial power spectra, the conversion formula to follow is then $r~=~v_{sc}/f$, where $v_{sc}$ is the spacecraft velocity with respect to Earth, and *f* is the sampling frequency of the in-situ instrument in question. In the right column plots we exploit this relation between the E- and F-region spectra, namely spatial scale, and plot the two spectra on the same *x*-axis. We normalize the density spectra by the rms of the clustering spectra. The reason for normalizing the quantities by a constant value is to make the slopes equivalent between the two graphed values. In return for sacrificing information regarding the relative power between the timeseries, we can now focus on the relative differences and similarities between the two.

The right-hand column of Fig. 2 shows that all three conjunctions feature a remarkably consistent shape-wise agreement between the F- and E-region power spectra, for small ($<8$ km) spatial scales, where individual features are at times reflected in both the E- and F-region spectra. On the other hand, they all differ drastically for the larger spatial scales, perhaps indicative of a characteristic transition scale. In all three F-region density spectra, we identify clear breakpoints, and in the case of the 22 May 2022 and 28 August 2021 conjunctions, the breakpoint occurs at the transition scale.

The matching of spectral index (slope) between the two different spectra has the principal consequence that there are certain turbulent quantities in the stochastic processes in both the bottomside- and the topside ionosphere that map, are observed simultaneously, and are approaching identical in the ideal case. The spectral index value that the regions agree upon tends to be in the dissipative regime, such as in Fig. 2, and in general, as we shall soon show. It is then tempting to conclude that the shape-wise close correspondence between the spectra seems *dependent* on the dissipative processes. For the scales said to be inertial in the F-region ($>8$ km), the E-region clustering spectra are consistently steeper than those of the F-region. It remains to be seen whether this disagreement for scales below the breakpoint scale could be outside an area of validity in the method. However, given that there is a special emphasis on the breakpoint-scale in the range of agreement (which should be purely physical), the disagreement in spectral index could be indicative of a characteristic non-mapping at larger scales.

**Figure 3:**
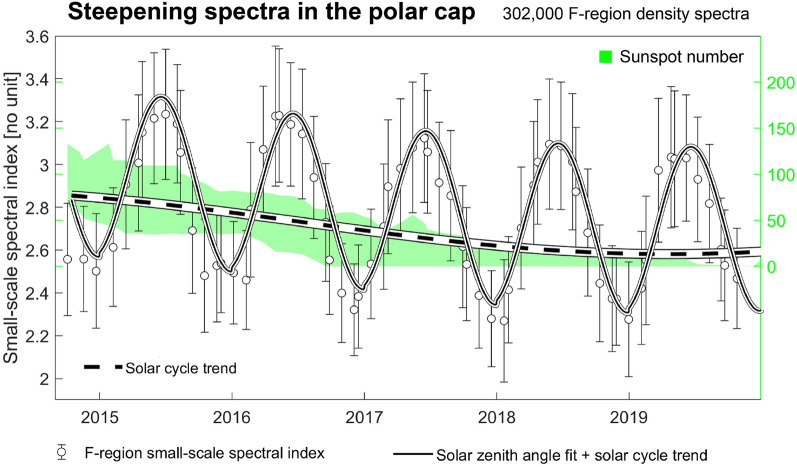
Long-term trends in density spectral steepening in the F-region polar cap. Circle datapoint represents the median small-scale spectral index value in a Carrington rotation in all steepening spectra observed poleward of $82^\circ$ mlat, with vertical errorbars corresponding to the variation (standard deviation) inside the Carrington rotation. A thick dashed black line shows the fit of Eq. (2), and a solid black line shows the fit of a sum of Eqs. (1) and  (2). Note that we here show absolute values for the spectral indices, meaning that increasingly positive values correspond to steepening. A green shaded area shows sunspot number. The extent of the shaded area corresponds to 5% and 95% percentile number of sunspots for each Carrington rotation.

Due to the lack of continuous Swarm 16 Hz density data coverage in 2021, 2022, conjunctions such as those in Fig. 2 are sparse. However, the above argument needs a bigger body of evidence to back it up. As mentioned, our ultimate goal is to complement the sparse set of conjunctions with a statistical comparison between the two datasets. However, before doing so, we must briefly discuss the nature of steepening F-region density spectra, *in the polar cap*, since density spectra from these two regions exhibit much of the same spectral shape (see Figure 2 in^9^). Figure 3 in the present paper shows each in black circle points the median small-scale spectral index for each solar 27-day rotation period (also called a Carrington rotations), for the entire polar cap. Errorbars denote upper and lower quartile distributions in each Carrington rotation. In addition we fit a predominantly seasonal function to the data. Here, we resort to a linear fit between the solar zenith angle at each measurement point and the spectral index^9^,

1 $$\begin{aligned} \alpha (Z) = a\times Z + b, \end{aligned}$$

*a* and *b* being parameters found through nonlinear least square minimizing of the functions. The solid black line in Fig. 3 is the average solar zenith angle in the polar cap (*Z*) multiplied by a slope *a*, and with an intercept *b*, with an additional solar cycle trend added:

2 $$\begin{aligned} \alpha (t) = \mu + \frac{3\sigma }{2}\sin \left( \frac{2\pi }{11 \text {years}}\times t + \theta _0 \right) , \end{aligned}$$

where $\mu$ and $\sigma$ are the median and standard deviation respectively, of the long-term 1-week median polar cap spectral index, *t* is the number of days elapsed since 0 January year 0, and $\theta _0$ is a phase shift which simply shifts the functional minimum to the end of 2019. To illustrate the real observed solar cycle trends, and to validate Eq. (2), we show with green shaded area in Fig. 3 mean sunspot number in each Carrington rotation, along the right *y*-axis. Although the dashed black line in Fig. 3 (Eq. 2) is fitted to the spectral index data, it manages to capture the long-term sunspot trends appreciably well. More to the point, the sum of Eqs. (1) and (2) does fit the F-region polar cap spectral index observations remarkably well, showing that spectral steepening (the index value, or slope) itself is highly seasonal in the F-region polar cap, with a clear but modest solar cycle trend.

**Figure 4:**
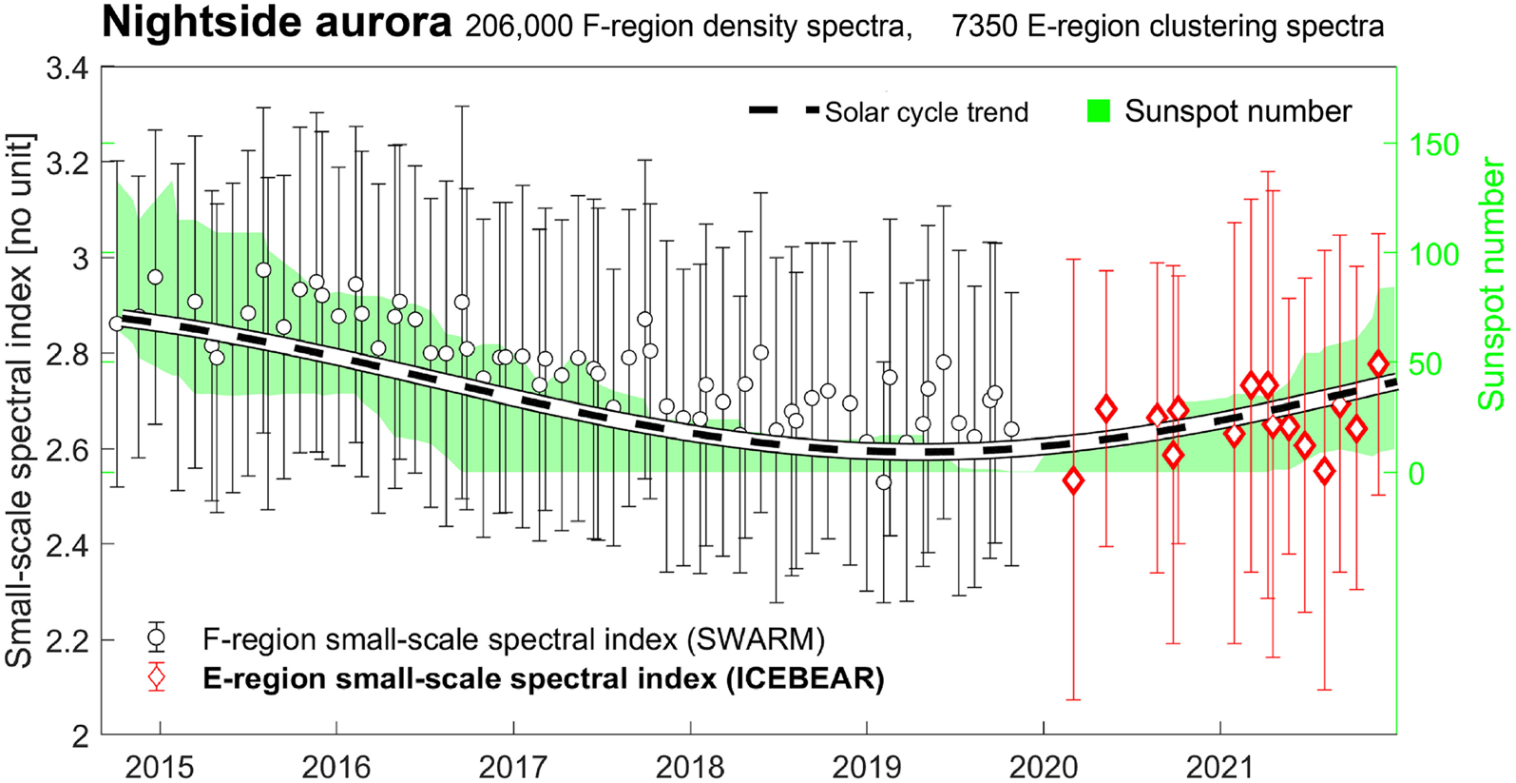
Long-term trends in density spectral steepening in the nightside aurora. The F-region and E-region datasets in aggregate for the auroral region, with Swarm A’s observations in black and icebear’s observations in red, both based on observations inside the nightside aurora (Fig. 1c). Each Swarm A-datapoint represents the median small-scale spectral index value in a Carrington rotation, with vertical errorbars corresponding to the variation (standard deviation) inside the Carrington rotation. Each icebear 3D-datapoint represents the slope of the mean spectrum found in each Carrington rotation, with errorbars denoting the variation (standard deviation) in the individual spectral indices for that period. The solar cycle trend is fitted using Eq. (2), for the icebear data (red) only. A green shaded area shows sunspot number. The extent of the shaded area corresponds to 5% and 95% percentile number of sunspots for each Carrington rotation.

**Figure 5:**
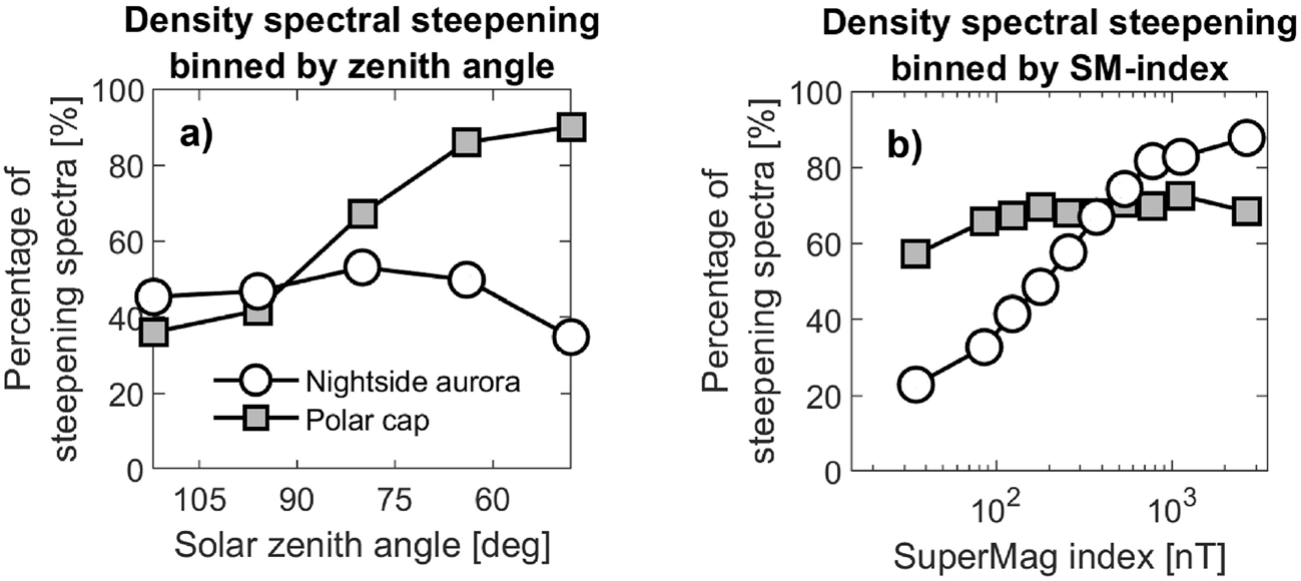
How F-region density spectra respond to solar zenith angle and auroral activity. Panel (**a**) shows the proportion of F-region density spectra that exhibit steepening in each solar zenith angle-bin, for the nightside aurora (circles) and the polar cap (squares). Panel (**b**) likewise shows the proportion of F-region density spectra that exhibit steepening in each sme-index-bin.

This is in stark contrast to the behaviour of the *auroral region* spectral indices (Fig. 4), which shows an equivalent long-term analysis applied to data from the nightside aurora (red box in Fig. 1c). Here, there are no discernible seasonal dependencies, but with roughly the same solar cycle trend. To be precise, any seasonal trend is lost in the errorbars in Fig. 4, which, as we prove in Fig. 5 below, represent day-to-day variation caused by changes in geomagnetic activity.

Before discussing the root cause of the trends displayed in Figs. 3 and 4, we must introduce the red datapoints in Fig. 4: the icebear 3D data. We collect 7350 E-region clustering spectra from the auroral region, occurring throughout 2020 (partially) and 2021. Figure 4 shows in red the median clustering spectrum for all spectra measured in a Carrington rotation. As mentioned, there is little direct overlap between the datasets, but the trends in sunspot number (green shaded area, Eq. 2 in dashed black line) provide a context, where the solar cycle fit can conceivably capture long-term trends in both datasets, though the Swarm-measured datapoints appear mostly slightly above the fit. The implication is that we are looking at the two different quantities as measurements of the same underlying quantity in nature, which in turn implies a field-aligned mapping of certain turbulent properties.

### Seasonality and geomagnetic activity trends

The reason for the observed seasonal trends in Fig. 3 is clear^31^. Strongly magnetized electrons are in effect frozen in Earth’s magnetic field. The motion of the ions then induces an ambipolar electric field, which serves to severely retard the otherwise fast ion diffusion. In the polar cap, during summer, a high E-region Pedersen conductivity shorts out the ambipolar electric field associated with F-region plasma irregularities, causing plasma to diffuse at the high ion perpendicular diffusion rate instead of the balanced ambipolar diffusion rate. On the other hand, during polar winter, the E-region conductivity can become negligible, leading to slow ambipolar diffusion. The periodic increase and decrease in irregularity decay is made manifest through the varying F-region small-scale spectral index as measured by Swarm A: when ambipolar diffusion speeds up during summer, F-region density spectra tend to steepen considerably, going from around 2.3 during winter, to around 3.2 during summer (Fig. 3). This shows that small-scale spectral index can effectively be used to measure the extent of seasonal changes in polar cap Pedersen conductivity.

Crucially, Pedersen conductivity in the polar cap is largely dependent on solar zenith angle. If we turn the situation around, then, we could surmise that the spectral indices of small-scale dissipating irregularities are inherently linked to changes in ionospheric Pedersen conductivity. This argument is supported by the general similarity between density spectra in the polar cap and in the nightside aurora, as we reported in a previous study: F-region density spectra from those two sectors show the same statistical properties, with, on average, more or less identical spectral shapes (see Figure 2 in^9^). Seeing as conductivity dynamics clearly are responsible for the steepening in the polar cap (Fig. 3), it is sensible to assume that E-region conductivity enhancements are likewise intimately connected to spectral steepening on display in the nightside aurora (Fig. 4). There, as alluded to, the ionosphere is usually in darkness, and ionization at E-region altitudes is due to high-energy particle precipitation^46,47^.

If the spectral steepening in the auroral region is largely driven by particle precipitation, we should see some clear dependencies on geomagnetic activity. We here turn to geomagnetic indices. The Supermag (sme) index is a recent auroral electrojet index^48^, which has been demonstrated to correlate well with total integrated nightside auroral power^49^. In Fig. 5, we present the response in the F-region density spectra to changing solar zenith angle and auroral activity. Panel (a) shows the dependency of F-region spectral steepening to changing solar zenith angle, corresponding to changes in solar EUV photoionization, for the nightside aurora and the polar cap. Panel (b) shows the dependency in the same quantity as a function of sme-index, for both regions. We see that while the proportion of steepening spectra in the polar cap increases dramatically as the zenith angle crosses 90$^\circ$ (the solar terminator), there is no such movement in the nightside auroral datapoints. In other words, EUV photoionization from the sun does not impact spectral steepening in auroral plasma. In Panel (b), however, the situation is opposite: polar cap density spectra do not steepen considerably as geomagnetic activity increases, while spectra from the auroral region undergo an even more dramatic steepening, from around 20% when the sme-index is below 70 nT, to around 90% for the most disturbed conditions (sme-index $>1000$ nT). As an aside, we mention here that solar zenith angle and the sme-index are not correlated. Solar zenith angle (at a fixed point on Earth) depends only on time, with clear diurnal and seasonal dependencies. The sme-index, on the other hand, measures the nightside Hall currents, and follow geomagnetic activity. However, the Russel–McPherron effect due to Earth’s dipole tilt will introduce a minor dependency between the two quantities^50^. The Russel-McPherron effect means that the equinoxes feature elevated geomagnetic activity^51^, which in itself should not introduce obvious solar zenith angle dependencies in the sme-index.

Going back to Figs. 3 and 4, we conclude that in each Carrington rotation in those figures, day-to-day variation in geomagnetic activity is the cause of the variation associated with the vertical errorbars. Moreover, geomagnetic activity variation has a much larger impact on the observations in the auroral region, where most high-energy particles are found, compared to the polar cap. Hence, the polar cap-errorbars in Fig. 3 are small, reflecting a reduced tendency for high-energy particles to cause E-region ionization, as is also shown in Fig. 5b.

### F-region vs. E-region

**Figure 6:**
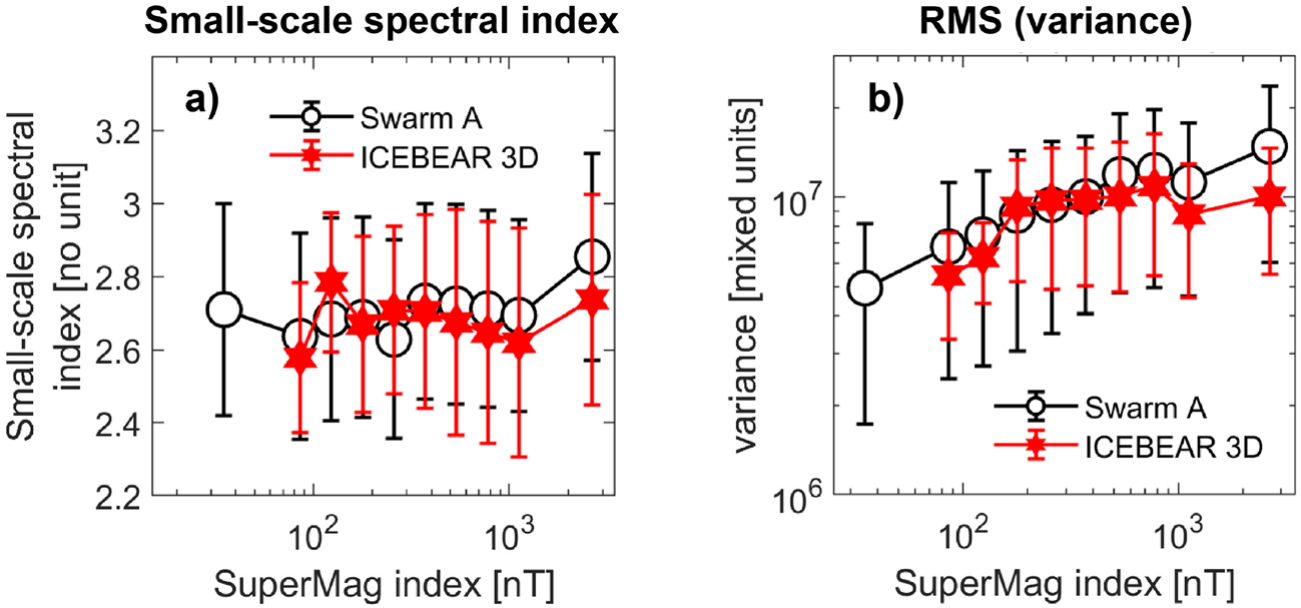
Spectral properties binned by geomagnetic activity, in the nightside aurora. Panel (**a)** shows the small-scale spectral index, while panel (**b)** shows the total integrated power (rms), both binned by sme-index, and both showing the F-region density spectra in black circles and the E-region clustering spectra in red filled hexagrams, with errorbars denoting upper and lower quartile distributions. The rms in panel (**b**) is obtained by integrating psd over a similar frequency/wavenumber interval for both instruments. It is integrated between 1 and 25 km for Swarm A, and between 1.5 and 25 km for icebear 3D.

The steep increase in the white circle points in Fig. 5b indicates that the overall steepening of F-region density spectra in the auroral region strongly depends on auroral activity being high, suggesting that the aurora, or particles associated with substorm injection events, are driving variability in the spectral properties there. What impact then does auroral activity have on the spectral *shapes* in both our datasets, the E-region clustering spectrum included? In Fig. 6, we show two spectral properties binned by the sme-index, for both datasets in the nightside aurora. Panel (a) displays the small-scale ($<8$ km) spectral index for the F-region density spectra (black circles) and the E-region clustering spectra (red hexagrams), from fitting spectral slopes to the mean spectra in each bin, and with vertical errorbars denoting the variability in the individual spectral indices. While we see that there is little change in small-scale spectral index across the interval, we note that the two datasets respond similarly to changing auroral activity: witness the common spike at the last sme-index bin. Moving on, Panel (b) displays the total density variation found in the F-region spectra (black circles), and the total clustering variation found in the E-region spectra, where we show median values for each sme-index bin, vertical errorbars representing upper and lower quartile distributions in each bin. Variance is here calculated as total integrated psd, also called root-mean-square (rms), where we integrate the spectra between 1 and 25 km for Swarm A, and between 1.5 and 25 km for icebear 3D (the smallest scale available for the clustering spectra is 1.5 km). Put simply, rms is the area under the graph showing psd, a quantity that is readily extracted from both the F- and E-region spectra. Note that the units along the *y*-axis are here arbitrary as the clustering rms has been shifted along the *y*-axis to facilitate comparison of the sme-index dependencies. This normalization is essentially a multiplication by a constant number, and so the shape of the two curves in Fig. 6 can be directly compared. Here we do see a clear response in the data to changing auroral activity. The sme-index dependency displayed by the F-region density variance is closely matched by the E-region clustering variance, down to the dip seen when the sme-index is around 1000 nT.

The close correspondence seen in Fig. 6 is remarkable, as the two databases do not overlap in time, and so single big events cannot be responsible for the features observed. The close correspondence in spectral index ($\sim -2.6$) is also broadly reflected in the conjunctions on display in Fig. 2, and in high-latitude steepening density spectra in general^9^. Such steep spectral indices are associated with irregularity dissipation or turbulent diffusion^13^. The common features for sme-index bins higher than 1000 nT that we observe in both panels of Fig. 6 could be caused by the equatorward portion of the auroral oval moving out of icebear’s field of view, thanks to the expansion of the oval with increasing geomagnetic activity. Since the morphology of the aurora differs between the poleward and equatorward boundaries of the auroral oval, observed spectral properties are likely to change.

## Discussion

The most natural explanation for the steepening density spectra in the auroral zone is the turbulent re-distribution of energy, through the growth of instabilities at some larger scales and the subsequent accelerated return to equilibrium^12,13,52^. This interpretation is strongly supported by Figs. 5b and 6b where both the proportion of steepening density spectra and the total density variance depend on geomagnetic activity. However, as is shown by Fig. 6a, the small-scale spectral index stays the same, in the classically denoted ‘dissipative regime’. A possible interpretation of the foregoing is that a shared driver, e.g., the magnetospheric substorm cycle, is driving the growth of turbulence in the E-region through an injection of high-energy particles. The turbulence subsequently enters a dissipative regime due to conductivity enhancements, enhancements that are indeed caused by the precipitating particles themselves^9,21,23,46^. The observed F-region density irregularities then largely depend on what is going on in the *E-region*.

Figures 2, 4 and 6 all agree: the E- and F-regions, and thus the entire ionospheric altitude column, are exhibiting remarkably similar small-scale turbulent properties. The behaviour on display in the three conjunctions in Fig. 2 is corroborated by the statistical aggregates, despite the fact that the latter do not overlap in time. The implication is that, given a conducting E-region, whenever a small-scale turbulent structure is observed in the F-region, it should also be observable in the E-region. Figure 2 clearly shows that this is true for scales as small as 1.5 km, fluctuation scales that are favourable for GNSS scintillations^53–57^. Our results then indicate that such radio scintillations could well originate in the E-region at high latitudes^58,59^.

Whereas the spectral agreement on smaller scales is apparent, the disagreement on the larger scales on display in Fig. 2 is an important result: the E-region spectra exhibit spectral indices in the dissipative regime for the entire scale interval, while the F-region spectra only do so below the breakpoint. Indeed, the initial spectral index (above the breakpoint) tends towards − 5/3 for both the polar cap and the auroral region^9^. We are then seeing a clear discrepancy for scales larger than the F-region breakpoint. The vertical mapping is explicitly dependent on both Hall and Pedersen conductivities^9^, conductivities that also cause the irregularity dissipation associated with the $\sim -2.6$ spectral index^31^. Based on this, we can speculate that conductivity dynamics are responsible for the large-scale discrepancy on display in Fig. 2, but the topic needs further study.

As mentioned, steepening density spectra are fundamentally similar in the polar cap and in the auroral region. However, Fig. 5 makes it clear that the two regions exhibit completely different drivers. The common feature is Pedersen conductance; in the polar cap conductivity enhancements are supplied by solar EUV photoionization while in the nightside aurora they are supplied by high-energy electron precipitation. Couple that with the F-region spectral breakpoints that are found on $\sim$km-scale, and we are led to hypothesize on the nature of the turbulent structuring we observe throughout the northern high-latitude ionosphere.

The gradient drift interchange instability is triggered by density gradients, and through charge separation and subsequent polarization electric fields, leads to a $\varvec{E}\times \varvec{B}$-drift that amplifies the density gradients that started it^3,31^. The growth rate of this instability favours small ($\sim ~1$ km) scales^3^, and is known through nonlinear simulations to cause a spectral breakpoint at scales between 2 and 3 km^60^. Moreover, that steepening polar cap density spectra at small scales are fundamentally similar to those found in the nightside aurora points to an instability mechanism that works independent of particle precipitation.

Regardless of which instability mechanism is responsible for the observed turbulent structuring, the close correspondence between the E- and F-region reported in the present study is testament to the role of conductivity dynamics in producing these instabilities. Although we do not have E-region observations from the polar cap, the vertical mapping of small-scale information between the E- and F-regions is ubiquitous in the auroral dataset at hand.

To summarize, by extensive analysis of in-situ F-region density spectra measured by Swarm A along with ground-based E-region clustering spectra measured by icebear 3D we have made several discoveries:

1. F-region density spectra are closely linked to E-region clustering spectra, both in spectral shape and in total spectral power. The two quantities stem from completely different analyses; the former is based on plasma density fluctuations in the topside ionosphere and the latter characterizes the tendency for 3-m FB waves to cluster in the bottomside ionosphere. As such, this finding is remarkable in itself. It shows that the ionospheric irregularity field exhibits similar statistical properties along the entire altitude column for the scales under consideration (between 1.5 and 25 km), and that the clustering of 3-m FB waves in the E-region closely follows the plasma density irregularity field.
2. Whereas auroral-zone irregularity power increases with geomagnetic activity, the spectral indices stay firmly in the classical dissipative regime—this is true for both the E- and F-regions. In the databases at hand, E-region ionization, irregularities, and irregularity dissipation are all ubiquitous, and difficult to separate. We interpret this to mean that the growth of turbulent structures in the auroral region tends to be accompanied by E-region ionization and the subsequent dissipation it causes. Taking the previous point into consideration, we note that this turbulent irregularity dissipation is working in tandem both in the E-region and topside F-region, and proves that the turbulent energy ledger maps vertically along the altitude column for the small scales.
3. The common trait for steepening density spectra in the polar cap and the nightside aurora is enhancements in Pedersen conductance, suggesting that E-region conductivity dynamics should be considered closely when discussing high-latitude plasma irregularities.

## Data Availability

icebear 3D echo data for 2020, 2021 is published with DOI 10.5281/zenodo.7509022. Data from the European Space Agency’s Swarm mission can be downloaded from the webpage https://swarm-diss.eo.esa.int/. Supermag data can be accessed at https://supermag.jhuapl.edu/mag/. Sunspot numbers are provided by SILSO, World Data Center-Sunspot Number and Long-term Solar Observations, Royal Observatory of Belgium, and can be accessed from http://www.sidc.be/silso/.
